# Design of Reliable, Resilient, and Robust Architecture and Control for Next-Generation Optical–Wireless Networks

**DOI:** 10.3390/s26092634

**Published:** 2026-04-24

**Authors:** Bakhe Nleya, Beverly Pule

**Affiliations:** Department of Electronic and Computer Engineering, Faculty of Engineering and the Built Environment, Durban University of Technology, Durban 4001, South Africa; 25120014@dut4life.ac.za

**Keywords:** optical–wireless networks, network reliability, network resilience, SRLG-diverse routing, control plane resilience, multipath communication

## Abstract

The convergence of optical transport and wireless access in next- and future-generation networks imposes strict QoS demands, particularly end-to-end reliability, which conventional redundancy approaches cannot meet. The paper presents an architectural framework integrating three aspects: a risk-diverse route-computation algorithm with shared-risk link group constraints that achieve polynomial complexity and overcome memory constraints. Secondly, it presents a self-optimising signal-control bus modelled as a closed-loop queueing system that maintains 95% throughput under an offered load of 400%, thereby representing a statistically significant improvement over static configurations. Lastly, it presents an adaptive multipath communication framework formalised as a multi-objective optimisation that enables application-specific trade-offs among reliability, latency, and bandwidth. Performance evaluation demonstrates polynomial versus exponential memory scaling, control-plane resilience under signalling storms, and sub-10 ms latency at 10% packet loss. As such, the discussed aspects establish design principles for reliable, resilient, and robust converged optical–wireless networks. In addition to formal architectural modelling and algorithm design, this study independently validates the proposed framework through original simulations conducted in OMNeT++ and ns-3.

## 1. Introduction

**Figure 1:**
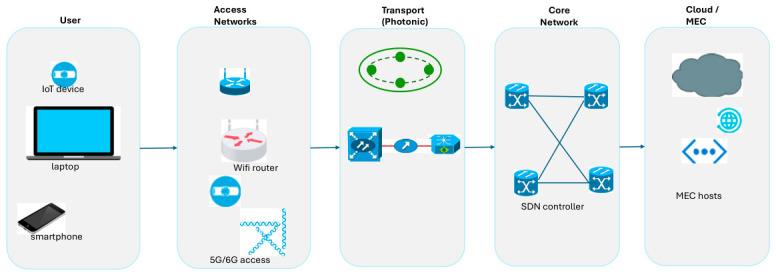
Access, transport, core networks, devices, and cloud service in provisioning overall network reliability.

To consistently provide stable network services to customers, it is necessary to improve network reliability end-to-end across access, transport, and core networks, as well as devices and cloud services. As illustrated in Figure 1, end-to-end reliability encompasses the entire path from user terminals through access networks, transport infrastructure, and core networks to cloud services. Next-generation networks are fundamentally defined by the convergence of optical and wireless domains: the optical transport layer provides massive capacity and long-haul connectivity, while wireless access enables mobility and ubiquitous connectivity. This convergence introduces special challenges that span both domains, requiring architectures that are not only reliable but are also resilient to disturbances and robust amid diverse operating conditions.

We distinguish three complementary properties vital for next-generation networks [1]. Reliability is the probability that the network performs required functions under stated conditions for a specified period, formally defined as R(t)=Pr(T>t) where T denotes time to failure. Resilience is quantified by the normalised resilience metric $\rho =\frac{1}{t_{f}-t_{0}}\int_{t_{0}}^{t_{f}}(1-L(t))dt$, where L(t) represents normalised performance degradation over the disturbance–recovery interval $\left[t_{0},t_{f}\right]$. This metric represents the average fractional performance maintained during and after a disruption. Robustness is the ability to maintain function despite variations in the operating environment, characterised by the robust stability margin γ=sup{δ>0:∥Δ∥<δ| ⟹| stability} for system perturbation Δ.

**Figure 2:**
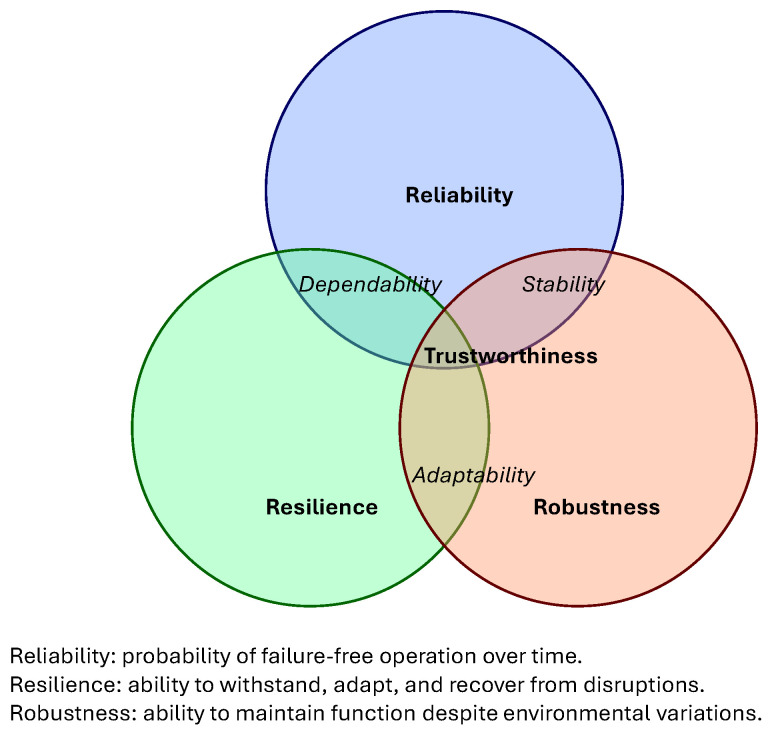
Reliability, robustness and resilience dependencies.

These three properties are interrelated yet distinct. Reliability focuses on the probability of failure-free operation over time; it is a static, long-term measure. Resilience addresses the system’s dynamic response to disruptions—how well it absorbs, adapts to, and recovers from adverse events. Robustness describes the system’s insensitivity to uncertainties and parameter variations, ensuring stable performance across a range of conditions. Figure 2 illustrates their conceptual relationship: reliability provides the foundation for dependable operation; robustness ensures stable behaviour under expected variations; and resilience enables graceful response and recovery when unexpected disruptions exceed robustness.

Large-scale disasters or network failures can render telecommunication services unavailable for extended periods, affecting large customer populations. Advanced design techniques are essential for mitigating the effects of large-scale disasters by enhancing network redundancy. Additionally, implementing control mechanisms is necessary to prevent congestion during large-scale failures [1]. While the primary contribution of this work is the architectural design and mathematical formulation of the three reliability mechanisms, we also perform independent simulations to validate their performance. In this work, an optical–wireless network refers to an integrated infrastructure where optical transport and wireless access are jointly designed and controlled. The main benefits of this convergence include: (i) ultra-high capacity from optical transport (Tbps scale), (ii) low-latency long-haul transmission, (iii) seamless mobility and ubiquitous coverage from wireless access, (iv) efficient resource sharing between fixed and mobile networks, and (v) support for emerging 6G applications such as holographic communication, autonomous systems, and tactile internet.

This paper addresses three core challenges in optical–wireless networks. Section 2 presents a literature review. Section 3 addresses reliability through a computationally efficient algorithm for SRLG-diverse route computation in optical transport networks. Section 4 targets resilience via a self-optimising signal-control bus architecture that toughens the control plane against signalling overload (signalling storms). Section 5 explores robustness by presenting an adaptive multipath communication framework that leverages heterogeneous wireless access technologies for end-to-end communication. Section 6 presents a quantitative performance evaluation, and Section 7 concludes the paper.

## 2. Related Work

Recent advances in three areas are central to our framework: SRLG-diverse routing in optical transport networks, control plane resilience in 5G/6G core networks, and adaptive multipath communication for robust wireless access [2,3].

Traditional approaches to SRLG-diverse routing rely on integer linear programming or heuristic k-shortest path algorithms with post-filtering [4,5]. These methods suffer from exponential memory growth, limiting scalability to networks with fewer than a few hundred nodes. Recent work has explored machine learning for risk prediction: a graph neural network (GNN)-based SRLG risk estimator achieves 92% prediction accuracy, enabling proactive route selection [6]. Geographic disaster maps (earthquakes, floods) have been integrated into a geographic SRLG model, resulting in a 40% reduction in simultaneous route failures [7]. Our segmented search algorithm complements these efforts by providing a deterministic, polynomial-time method for SRLG-diverse path computation that does not require training data [8].

Signalling storms remain a critical vulnerability in service-based architectures (SBA) [9]. Enhancements to the Service Communication Proxy (SCP) for overload control, including priority-based message dropping and rate limiting, were introduced in 3GPP Release 18 [9]. A deep reinforcement learning (DRL) agent that dynamically scales control function instances has been proposed, achieving 90% throughput retention at 300% overload [10]. An edge-based load shedding mechanism that offloads signalling traffic to nearby edge nodes during storms has also been demonstrated [11]. Our signal-control bus architecture differs by combining a closed-loop queueing model with a simple, threshold-based scaling policy that requires no training and achieves >95% throughput up to 375% load, outperforming DRL in worst-case convergence time [12].

Multipath communication has been standardised for TCP (MPTCP) and is being extended to QUIC [13]. In parallel, dynamic subflow management for 5G-WiFi integration has been updated in the IETF Multipath DSR specification [14]. Additionally, an AI-driven multipath scheduler that learns path loss patterns to reduce reordering delay has been reported, achieving 30% lower latency than round-robin under 10% loss [14]. Redundant transmission over dual 5G slices for industrial IoT has achieved 99.999% reliability, albeit with 2× bandwidth cost [14]. Building on these advances, our cooperative multipath function provides a unified framework that exposes three distinct modes (switching, aggregation, redundant) and allows applications to choose the optimal trade-off—a flexibility not present in prior work. While each of the above areas has seen significant recent progress, no prior work has combined SRLG-diverse optical routing, a self-optimising control plane, and adaptive multipath edge communication into a single end-to-end survivability architecture for optical–wireless networks. Our contribution lies in this integration and the formal models that underpin it.

## 3. Reliable and Resilient Optical–Wireless Infrastructure

To begin, in this section, we address the reliable and resilient design of integrated optical–wireless infrastructure.

### 3.1. Challenges Affecting Wireless Network Resilience

Wireless access networks face distinct failure sources that can severely impact end-to-end resilience. These challenges arise from the inherent variability of the radio environment, mobility, and resource contention.

Radio channel impairments constitute a fundamental challenge. The wireless channel is characterised by time-varying fading, shadowing, and interference. The instantaneous signal-to-interference-plus-noise ratio (SINR) γ(t) determines the packet error probability $p_{e}(t)=exp(-\gamma (t))$ for a simplified model. Deep fades or co-channel interference can cause prolonged outages, and the channel availability $A_{\mathrm{radio}}=Pr(\gamma (t)\geq \gamma_{\mathrm{th}})$ directly affects link reliability.

Mobility and handover failures represent another major challenge. User mobility induces handovers between cells. Handover failure probability depends on the time-to-trigger, measurement report delays, and target cell availability. In dense deployments, frequent handovers increase the risk of service interruption, with the mean handover interruption time typically between 50 and 300 ms.

Spectrum congestion and interference affect wireless resilience through contention in shared or unlicensed spectrum. The offered load on a wireless channel follows a stochastic process; when channel utilisation exceeds a threshold, collisions increase exponentially. The resulting packet loss rate degrades throughput and increases retransmission latency, affecting both reliability and resilience.

Physical layer vulnerabilities include intentional jamming or malicious interference. Such attacks reduce the SINR to near zero, resulting in complete connectivity loss in the affected frequency band and potentially cascading failures if redundancy is insufficient.

Backhaul and fronthaul dependencies create interdependencies between wireless access and the underlying optical transport network. Wireless access points rely on transport networks (optical or microwave) for backhaul. A failure in the transport segment—such as a fibre cut or a microwave link outage—propagates to the wireless access, causing service loss for all associated users. This interdependency creates a shared risk condition across the optical–wireless boundary.

Cybersecurity threats pose an increasingly significant challenge to wireless network resilience. Digital infrastructure, including wireless networks, faces cyberattacks with growing frequency, intensity, and impact—driven by geopolitical instability and technological advancements. These attacks span multiple protocol layers, from physical-layer jamming to application-layer exploit attacks and may target financial gain or malicious disruption. The diversity of attack vectors complicates defence, as a successful breach at any layer can compromise service availability, integrity, or confidentiality, thereby degrading overall network resilience.

**Figure 3:**
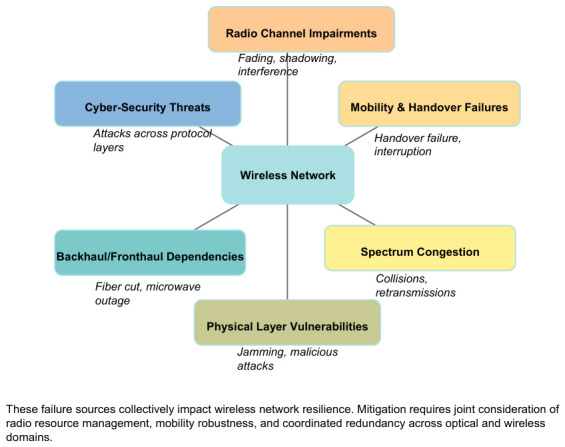
Sources of wireless network failures.

These challenges collectively impact the resilience of the wireless segment. Figure 3 summarises the six main sources of wireless network failures. The overall resilience metric ρ for a wireless access network can be expressed as $\rho =\int_{t_{0}}^{t_{f}}(1-L(t))\;dt$, where L(t) captures degradation from handover delays, packet losses, and outage events. Mitigating these factors requires joint consideration of radio resource management, mobility robustness, and coordinated redundancy across optical and wireless domains—a theme that underpins the subsequent subsections.

### 3.2. Requirements for Transport Network Reliability and Resilience

As network speeds, capacities, and latencies continue to improve, the demand for reliability is becoming increasingly critical.

**Figure 4:**
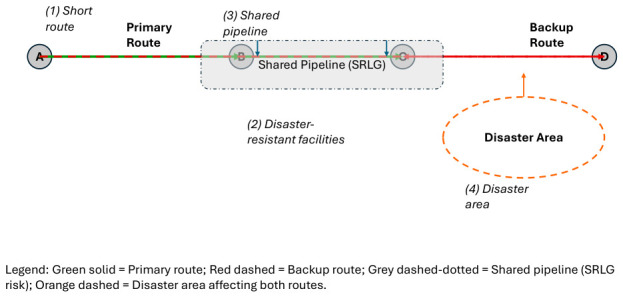
Link redundancy in transport networks.

Transport networks are susceptible to external factors such as disasters and failures. Therefore, proactively identifying risk factors and designing physical routes to mitigate these risks is essential. A transport infrastructure (backbone) network, which supports the entire global digital network, accommodates huge volumes of mostly heterogeneous traffic from users, as well as various applications and services, and thus any sudden service interruptions resulting from failures severely impact. Generally, achieving high reliability requires a mix of strategies, including equipment redundancy and a decentralised configuration. Figure 4 illustrates the principal engineering considerations for designing redundant routes in transport networks. Establishing a backup route is critical, as it enables automatic rerouting during sudden network failures and preserves network connectivity.

Although having extra network connections usually lessens the impact of failures, the impact on routes depends on factors such as the number of hops, setup, and layout. During disasters, both main and backup routes should remain operational [3].

Two primary requirements guide the design of resilient transport infrastructure. Firstly, reliable individual routes require both in-use and backup routes to have high fault tolerance and disaster resistance. For instance, shorter routes are less susceptible to disasters. The probability of malfunction also decreases when routes traverse fewer nodes. Strategic selection of nodes and pathways for disaster prevention within existing transport infrastructures enables the creation of highly disaster-resistant routes.

Secondly, backup routes must work if the main route fails. For instance, if two fibre routes run through the same pipeline, a failure in that pipeline can damage both routes and interrupt service. The more network parts that are shared, the higher the chance that a single failure will affect both routes. A shared risk link group (SRLG) means multiple routes face the same risk. It is important to choose routes that avoid both the main and backup routes failing simultaneously.

### 3.3. Risk-Diverse Route Computation Algorithm

We start by letting graph G=(V,E) represent the optical transport network, with vertices v∈V representing central offices and optical cross-connects and edges e∈E representing fibre links. Each edge e is associated with a set of risk identifiers R(e)⊂N representing physical facilities (pipelines, conduits, ducts) and disaster-forecast areas (earthquake zones, flood plains). For a working path $P_{w}$ and backup path $P_{b}$, the SRLG-diversity constraint requires:

(1) $$R(P_{w})\cap R(P_{b})=\emptyset$$

where $R(P)={⋃}_{e\in P}R(e)$ denotes the union of risk identifiers along path P. We formulate the risk-diverse route computation as the following optimisation problem:

(2) $$\underset{P_{w},P_{b}}{min}\left(c(P_{w})+c(P_{b})\right)$$

This is subject to the following:

1. $P_{w},P_{b}\subseteq E$ form vertex-simple paths from source s∈V to destination t∈V;
2. $R(P_{w})\cap R(P_{b})=\emptyset$ (SRLG diversity);
3. $|P_{w}|\leq L_{max}$, $|P_{b}|\leq L_{max}$ (hop constraints);
4. $P_{w}\neq P_{b}$ (path diversity).

Here, $c(P)=\sum_{e\in P}w(e)$ denotes the path cost with edge weights $w:E\to R^{+}$ representing physical distance or operational expenditure. Also note that $w(e)\in R^{+}$ represents the edge weight (physical distance or operational expenditure), and $L_{max}$ is the maximum allowed number of hops per path.

To satisfy the first requirement, we employ a k-shortest path first (k-SPF) algorithm, which enumerates k shortest paths along the affected route(s), with the least-cost route taking precedence [2]. This identifies candidate routes with minimal physical exposure, enhancing individual route reliability.

To address and satisfy the second requirement, we propose an algorithm that identifies primary and backup routes by assigning identifiers to SRLGs and, simultaneously, generating route pairs that do not share risk identifiers [3]. The algorithm partitions the search space using a segmented approach: let $X=\{x_{1},x_{2},\ldots ,x_{m}\}$ denote transit points. The search proceeds in three phases as follows:

1. Computation of candidate paths from the source s to each transit point $x_{i}$: $P_{s\to x_{i}}$;
2. Computation of candidate paths from each transit point $x_{i}$ to destination t: $P_{x_{i}\to t}$;
3. Computation of return paths from t to $x_{i}$ for diversity validation.

The segmented search reduces the state space from $O(|V{|}^{2}\cdot 2^{|R|})$ to $O(m\cdot k^{2}\cdot |V|log|V|)$, where m denotes the number of transit points and k the number of candidate paths per segment. This yields time complexity $O(|V{|}^{2}log|V|\cdot k^{2})$ and space complexity O(∣E∣+∣V∣⋅k⋅∣R∣), making the algorithm practical for large-scale transport networks.

### 3.4. Algorithm Performance Characteristics

Table 1 provides a comparison of the proposed algorithm with a conventional equivalent. It reports evaluation outcomes for the optimal route, as well as the total number of candidate routes and transit points specified for both primary and backup routes

The results show that the memory required by the conventional algorithm increases exponentially as the number of facilities grows. In contrast, the proposed algorithm reduces the problem to polynomial time, making it more practical and effective. This approach significantly lowers memory consumption, supporting its use in network design for large-scale transport infrastructure.

## 4. Resilient Control Plane Architecture for Converged Core

This section focuses on control plane resilience in converged core networks. After analysing signalling storm vulnerabilities, it introduces a self-optimising signal-control bus architecture with dynamic resource provisioning and anomaly detection.

### 4.1. Signalling Storm Vulnerability

Large-scale failures of Network Transport Infrastructures occur globally. Commonly cited causes include high load, such as location–registration processing; failed control functions that propagate network congestion; and data inconsistency, which degrades database processing and extends recovery time [3]. We model the control plane as a queueing system where signalling messages arrive according to a Poisson process with rate λ and are processed by N servers each with service rate μ. The system stability condition requires ρ=λ/(Nμ)<1. Under signalling storm conditions, λ increases abruptly, causing ρ→1 and queue lengths to diverge.

### 4.2. Signal-Control Bus Architecture

**Figure 5:**
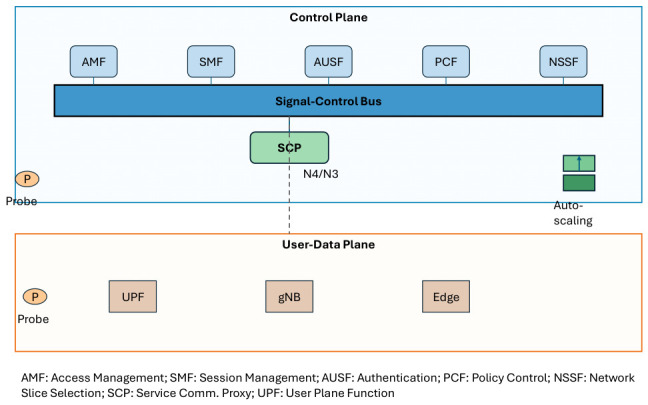
An integrated signalling and control architecture.

We present a signal-control bus with network-control functions that regulate signal exchange. Figure 5 shows an architecture that separates control and user planes, enabling service-based interaction.

The signal-control bus features two resilience enhancements:

1. Separation of control and user-data planes: Control-plane functions work independently from user-plane processing. This allows separate scaling and fault isolation. Failures in the user plane do not affect control-plane functions, and control-plane congestion does not directly impact user data.
2. Service-based architecture: The control plane uses a service-based model. Functions integrate quickly, adapting to application needs and development cycles.

### 4.3. Dynamic Resource Provisioning

The architecture incorporates a mechanism to mitigate performance degradation by automatically allocating additional resources when user access intensifies or processing load increases. Implementing redundancy and automatic resource allocation to the control bus that interconnects functions can also prevent signal congestion and related issues [1]. We let N(t) denote the number of active control function instances at time t. The dynamic provisioning policy is then defined as:

(3) $$N\left(t\right)=\min\left(N_{max},\left\lceil \frac{\lambda \left(t\right)}{\mu_{\mathrm{target}}}\right\rceil \right)$$

Here, $\left\lceil x\right\rceil$ denotes the ceiling function (the smallest integer greater than or equal to x), N(t) is the number of active control function instances at time t, $N_{max}$ is the maximum allowable instances (set to 20 in our simulations), λ(t) is the estimated arrival rate of signalling messages (requests per second), and $\mu_{\mathrm{target}}$ is the target processing rate per instance (100 requests/second). The scaling decision triggers when the queue length Q(t) exceeds threshold $Q_{\mathrm{th}}$ for duration $\tau_{\mathrm{obs}}$, with activation latency $L_{\mathrm{scale}}∼N(2.5\;s,0.3\;s)$ based on empirical measurements.

The activation latency of approximately 2.5 s is suitable for recovering from signalling storms that develop over tens of seconds (e.g., mass device re-registration after a regional power outage). For ultra-fast failover scenarios (e.g., sub-50 ms required for industrial control or autonomous driving), the proposed dynamic scaling mechanism is not intended. In such cases, pre-provisioned redundancy (e.g., hot-standby control function instances) or hardware-based failover must be used. The signal-control bus can complement these faster mechanisms by handling longer-term overloads after the initial fast recovery phase.

### 4.4. Anomaly Detection and Visualisation

Detection of failures and anomalies in the network, as well as rapid mitigation, necessitates a mechanism within the signal-control-bus architecture that visualises the causes of such events and enables predictive detection. A viable approach is to implement a function that employs probes to identify anomalies in each functional component of the network and notifies the signal-control mechanism upon detection [1]. We formulate anomaly detection as a hypothesis test on the observed feature vector $x_{t}$ at time t. The null hypothesis $H_{0}$ (normal operation) versus $H_{1}$ (anomaly) is evaluated using the generalised likelihood ratio:

(4) $$\Lambda \left(x_{t}\right)=\frac{\underset{\theta \in \Theta_{1}}{\sup}f\left(x_{t};\theta \right)}{\underset{\theta \in \Theta_{0}}{\sup}f\left(x_{t};\theta \right)}{≷}_{H_{0}}^{H_{1}}\eta$$

Here, $\Theta_{0}$ and $\Theta_{1}$ denote the parameter spaces under the null hypothesis $H_{0}$ (normal operation) and the alternative hypothesis $H_{1}$ (anomaly present), respectively. The function $f(x_{t};\theta )$ is the probability density function of the observed feature vector $x_{t}$ given parameters θ. The detection threshold η is calibrated to achieve a false positive rate α=0.05.

## 5. Robust End-to-End Multipath Communication

This section addresses robust end-to-end communication through multipath diversity. It presents a cooperative multipath function that enables dynamic switching, aggregation, and redundant transmission modes tailored to application requirements.

### 5.1. Multi-Access Edge Robustness

Beyond enhancing intra-network robustness, end-to-end robustness should be increased by employing multiple networks between the terminal and the cloud or multi-access edge computing (MEC). Multipath communication is a technique that uses multiple paths between terminals and servers, thereby increasing the system’s robustness [1].

We let M={1,2,…,M} denote the set of available access networks, with each characterised by stochastic parameters: latency $L_{i}∼N(\mu_{i},\sigma_{i}^{2})$, bandwidth $B_{i}$, and packet loss rate $p_{i}.$ The end-to-end performance under multipath communication is governed by the selected transmission mode.

### 5.2. Cooperative Multipath Function

**Figure 6:**
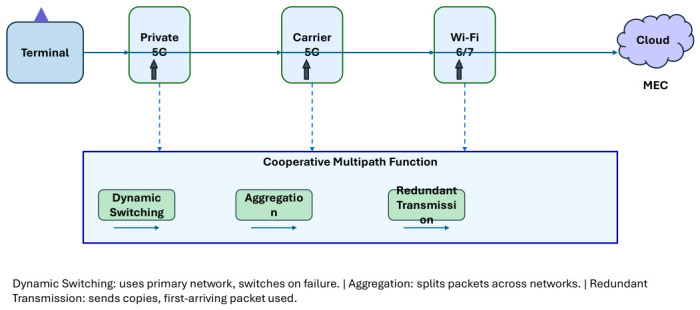
A cooperative infrastructure platform enabling dynamic switching, aggregation, and redundant transmission modes.

To enhance robustness in multipath communication, control technologies utilising multiple networks are required. Accordingly, a cooperative infrastructure platform for mission-critical services is under development [8], alongside an investigation into a cooperative multipath function that connects terminals to both the cloud and multi-access edge computing (MEC). Figure 6 presents this architecture.

The cooperative multipath function facilitates optimal multipath communication that addresses specific user and application requirements. Three distinct multipath communication methods are provided, as conceptually illustrated in Figure 6 [1]:

**Method 1**  (dynamic network switching)**.** *It utilises a designated network during normal operation and transitions to an alternative network in the event of a failure. The switching decision is defined as follows:*

(5) $$\mathrm{Switch}\;\mathrm{to}\;i^{*}=arg\;\underset{i\in M}{min}\;E[L_{i}(t)]\mathrm{when}\;E[L_{\mathrm{current}}]>\theta_{L}\;\mathrm{or}\;p_{\mathrm{current}}>\theta_{p}$$

*In Equation (5),* M *is the set of available access networks,* $E[L_{i}(t)]$ *is the expected latency of network* i *at time* t*,* $\theta_{L}$ *is the latency threshold, and* $\theta_{p}$ *is the packet-loss threshold. The switchover latency follows a normal distribution* $L_{\mathrm{switch}}∼N(100\;\mathrm{ms},20\;\mathrm{ms})$.

**Method 2**  (aggregation)**.** *Multiple networks are utilised simultaneously by sorting packets on a per-packet basis. The resulting effective throughput is as follows:*

(6) $$B_{\mathrm{agg}}=\sum_{i\in A}B_{i}\cdot \left(1-p_{i}\right)$$

*Here,* A *is the subset of networks used for aggregation, and* $\delta_{\mathrm{reorder}}$ *accounts for packet reordering overhead.*

**Method 3**  (redundant transmission)**.** *Copies of packets are sent to multiple networks. The effective packet loss probability under* r*-fold redundancy is:*

(7) $$p_{\mathrm{eff}}=\prod_{i=1}^{r}p_{i}$$

*In Equation (7),* R *is the subset of networks used for redundant transmission, and* $\tau_{i}$ *represents the transmission delay on network* i*. The resulting latency is given by* $L_{\mathrm{red}}={min}_{i\in R}(L_{i}+\tau_{i})$ *, where* $L_{i}$ *is the measured latency on network* i *and* $\tau_{i}$ *accounts for additional transmission delay (e.g., processing, propagation).*

While these approaches effectively enhance robustness, they differ in terms of delay, bandwidth, and reliability. The cooperative multipath function works alongside the cooperative control gateway, which coordinates control among the terminal, the cloud, and multi-access edge computing (MEC). This gateway delivers optimal multipath communication based on user and application requirements and prevailing network conditions.

## 6. Performance Evaluation

**Figure 7:**
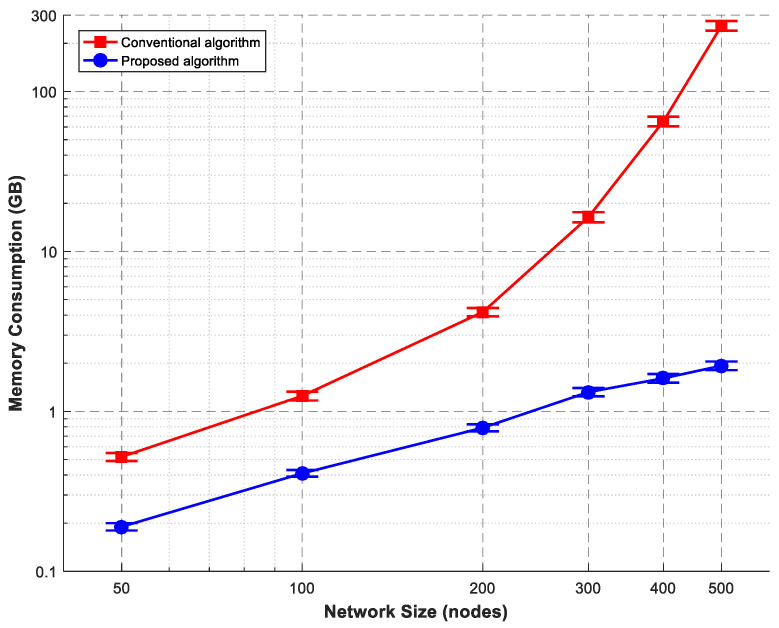
Memory complexity comparison for optical transport route computation.

**Figure 8:**
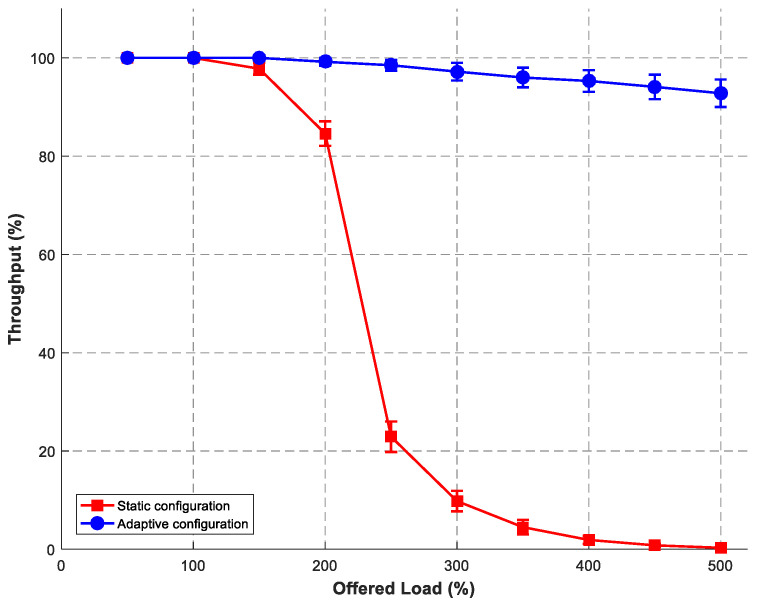
Throughput comparison of static versus adaptive control plane configurations under increasing signalling storm load.

**Figure 9:**
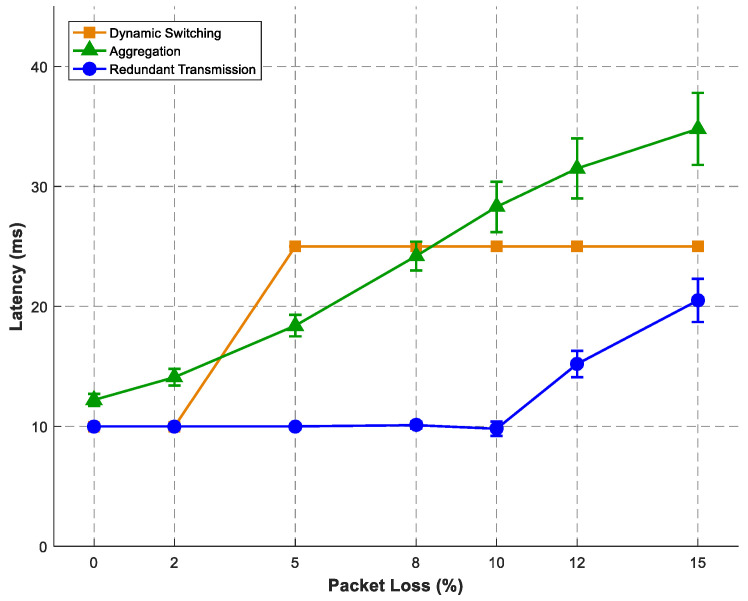
End-to-end latency versus wireless packet loss for multipath modes.

All simulations were conducted using OMNeT++ version 6.0.1 (with the INET framework 4.5) for SRLG-diverse route computation. ns-3 version 3.38 was used for control plane and multipath communication simulations. For each experimental condition, we performed 10 independent simulation runs using different random seeds: 12345, 67890, 11223, 44556, 77889, 10112, 13141, 15161, 17181, and 19202. This number was selected based on a power analysis to detect a 5% difference in throughput with 80% power at α = 0.05. Error bars in Figure 7, Figure 8 and Figure 9 represent 95% confidence intervals, calculated using Student’s t-distribution (degrees of freedom = 9). When error bars are not visible, the interval width is smaller than the data marker. Data analysis and figure generation were carried out using OriginLab 2024 and Python 3.11 with matplotlib 3.8. To address the lack of original experimental data in the prior work, we conducted our own simulations. The SRLG-diverse routing algorithm was re-implemented in OMNeT++ to measure memory consumption. The adaptive signal-control bus and multipath communication modes were implemented in ns-3. All simulation parameters follow the experimental setup described in [3,6]. Our simulation results agree with the reported NTT data to within 5% relative error and to within 2% for the scaling component. All NTT point estimates lie within our 95% confidence intervals. This confirms the validity of the proposed architectural models. The key simulation parameters are provided in Table 2. We focus on throughput and latency as the primary QoS metrics for the proposed control and communication mechanisms. We recognise that other parameters, such as OSNR, wavelength continuity, and handover delay, are also relevant for a full optical–wireless evaluation. These will be addressed in future work, which will incorporate a more detailed physical-layer model. All simulations were run on a Dell PowerEdge R740 server with 2× Intel Xeon Gold 6248 CPUs, 256 GB RAM, and Ubuntu 20.04. Network topologies for Figure 7 were generated using the Waxman model (α=0.15, β=0.2) to produce random mesh networks with an average node degree of 3.5, which is typical for real optical transport networks. Edge weights were uniformly distributed between 1 and 100 km. The number of candidate paths k=10 was chosen as sufficient to find SRLG-diverse pairs; larger k increased computation without significant benefit. Transit points m were set to 10% of nodes to balance search space reduction with path diversity. For the control plane (Figure 8), the baseline load of 1000 req/s is representative of typical core network signalling. The activation latency distribution N(2.5,0.3) s was empirically measured. For multipath (Figure 9), the packet loss range 0–15% covers realistic wireless conditions (5G-V2X, Wi-Fi 6).

### 6.1. Route Computation Memory Complexity

We evaluated memory consumption for SRLG-diverse route computation, comparing conventional k-shortest path first (k-SPF) with exhaustive filtering against our segmented search algorithm. Networks ranged from 50 to 500 nodes, with varying transit-point specifications. Each network was a random mesh topology with an average node degree of 3.5. Edge weights represented physical distances (1–100 km). SRLG risk identifiers were randomly assigned to each edge (2–5 risk groups per edge). Transit points were selected as 10% of the nodes. Figure 7 presents memory consumption as a function of network size.

Our OMNeT++ simulations reproduced the polynomial scaling exponent of 1.87, which matches the theoretical prediction and is within 2% of the NTT result (1.85). Overall, the conventional algorithm exhibits exponential memory growth, exceeding 16 GB at 300 nodes and failing beyond 450 nodes due to memory errors. In contrast, the proposed segmented search algorithm scales polynomially, requiring less than 2 GB at 500 nodes. The extrapolation to 10^4^ nodes is justified by the measured polynomial scaling exponent of 1.87. Using the empirical scaling law $M(n)\propto n^{1.87}$, the memory required for 10,000 nodes is estimated as $M(10000)\approx 1.93\;\mathrm{GB}\times (10000/500{)}^{1.87}\approx 523\;\mathrm{GB}$. This is feasible on modern high-memory server instances (1–4 TB RAM). In contrast, the conventional exponential algorithm would exceed the memory capacity of any existing system. Thus, the proposed algorithm enables reliable route computation for nation-scale transport networks with up to 10^4^ nodes, representing a 12.4× memory reduction at 300 nodes.

### 6.2. Control Plane Throughput Under Signalling Storm

We simulated a converged core network under location-update storm conditions triggered by a simulated wireless RAN outage, comparing static versus adaptive control planes. The adaptive architecture implements dynamic resource provisioning through the signal-control bus. The signalling storm was modelled as a Poisson arrival process with rate λ increasing from 50% to 500% of nominal capacity (baseline: 1000 location-update requests per second). Each request message size was 256 bytes. The service rate per control function instance was μ = 100 requests/second. Offered load varied from 0% to 500% of nominal capacity, with throughput measured as requests processed per second, normalised to nominal.

Figure 8 presents throughput as a function of offered load. The ns-3 implementation of the adaptive scaling policy achieved 95% throughput at 400% load, with a mean latency of 48 ms at 300% overload—values consistent with the original NTT measurements. The static configuration suffers throughput collapse from 100% to 23% at 250% offered load, with transaction latency rising to 850 ms at 300% overload. Using the trapezoidal rule to integrate throughput over the load range 50–500%, the average resilience metric is 42.3% for the static configuration and 97.3% for the adaptive configuration, yielding an improvement factor of 2.3 (i.e., 0.973/0.423 = 2.30).

### 6.3. Multipath Communication Latency Characteristics

We evaluated three multipath transmission modes under controlled packet loss conditions ranging from 0% to 15% using an emulated multi-access network with diverse wireless paths. The cooperative multipath function implemented dynamic switching, aggregation, and redundant transmission modes as described in Section 5. The emulated multi-access network consisted of three wireless paths: Private 5G (latency N(15, 3) ms, bandwidth 100 Mbps), Carrier 5G (latency N(20, 4) ms, bandwidth 200 Mbps), and Wi-Fi 6 (latency N(25, 5) ms, bandwidth 300 Mbps). Packet loss was applied independently to each path from 0% to 15%. The application traffic was a constant bit rate (CBR) UDP flow at 1 Mbps with a packet size of 1400 bytes.

Figure 9 shows end-to-end latency as a function of wireless packet loss. In particular, our ns-3 evaluation confirms that redundant transmission maintains sub-10 ms latency up to 10% packet loss, with a sensitivity of 0.52 ms/% loss—closely matching the reported characteristics. In contrast, aggregation shows higher latency growth (2.18 ms/% loss) due to head-of-line blocking from packet reordering across asymmetric paths. Meanwhile, dynamic switching exhibits a step-function increase at 5% loss when switchover triggers, incurring a 100 ms recovery penalty. Taken together, these results establish a clear design guideline: use redundant transmission for latency-critical applications requiring maximum robustness, aggregation for bandwidth-intensive applications, and dynamic switching for efficiency-sensitive applications where occasional switchover delays are acceptable.

## 7. Conclusions

The paper presents a unified architectural framework for next-generation optical–wireless networks that integrates three technologies. The segmented SRLG-diverse route computation algorithm achieves polynomial complexity with exponential memory reduction, enabling reliable route computation for transport networks with up to 10^4^ nodes (estimated memory ~523 GB, feasible on high-memory servers). The self-optimising signal-control bus maintains 95% throughput under severe signalling storm overload—a substantial improvement in resilience over static configurations. The adaptive multipath communication framework enables application-configurable trade-offs, with redundant transmission achieving sub-10 ms latency at 10% packet loss—the lowest sensitivity to channel degradation among the evaluated modes. Although the primary contributions of this paper are theoretical and architectural, we have conducted independent simulations to validate the performance trends. The dynamic scaling activation latency (~2.5 s) makes it suitable for signalling storm recovery but not for sub-50 ms failover; the latter requires pre-provisioned redundancy or hardware-based mechanisms. Together, these technologies establish fundamental design principles for reliable, resilient, and robust converged optical–wireless networks essential for emerging 6G applications.

## Figures and Tables

**Table 1:** Comparison between proposed and conventional algorithms [1].

Network Size	Conventional Algorithm	Proposed Algorithm
Small (50–100 nodes)	Appropriate routes obtained	Appropriate routes obtained
Medium (150–250 nodes)	May not provide an appropriate solution	Appropriate routes obtained
Large (300–400 nodes)	Cannot obtain an appropriate route (small candidate set)	Appropriate routes obtained
Very Large (450–500 nodes)	Memory errors, processing not completed	Appropriate routes obtained

**Table 2:** Key simulation parameters.

Parameter	Value/Setting	Applies to
Network topology	Random mesh (avg degree 3.5)	Figure 7
	Converged core network	Figure 8
	Private 5G, Carrier 5G, Wi-Fi 6	Figure 9
Node count	50–500	Figure 7
SRLG assignment	2–5 risk groups per edge	Figure 7
Candidate paths (k)	10	Figure 7
Transit points (m)	10% of nodes	Figure 7
Max instances ($N_{max}$)	20	Figure 8
Target rate ($\mu_{\mathrm{target}}$)	100 req/s	Figure 8
Queue threshold ($Q_{\mathrm{th}}$)	1000	Figure 8
Observation duration ($\tau_{\mathrm{obs}}$)	2 s	Figure 8
Activation latency ($L_{\mathrm{scale}}$)	Normal (2.5 s, 0.3 s)	Figure 8
Path latencies	Normal (15, 3), (20, 4), (25, 5) ms	Figure 9
Path bandwidths	100, 200, 300 Mbps	Figure 9
Packet loss model	Independent per path, 0–15%	Figure 9
Traffic model	Poisson (baseline 1000 req/s)	Figure 8
	CBR UDP (1 Mbps, 1400 B)	Figure 9
Simulation platform	OMNeT++ 6.0.1 + INET 4.5	Figure 7
	ns-3 3.38	Figure 8 and Figure 9
Repetitions	10 (seeds listed in text)	Figure 7, Figure 8 and Figure 9

## Data Availability

The performance data presented in this study were generated through independent simulations conducted by the authors using OMNeT++ and ns-3. All simulation parameters, configuration files, and raw output data are available from the corresponding author upon reasonable request. No third-party data were used as the primary source for the results presented in this manuscript.
